# Novel method for the quantification of rosette area from images of *Arabidopsis* seedlings grown on agar plates

**DOI:** 10.1002/aps3.11504

**Published:** 2022-12-08

**Authors:** Anna Knapp, Jordan Stefani, Ella Katz, Arnold J. Bloom

**Affiliations:** ^1^ Department of Plant Sciences University of California Davis, One Shields Ave. Davis California 95616 USA

**Keywords:** automated measurement, diversity panel, high‐throughput phenotyping, image analysis, leaf area, Python

## Abstract

**Premise:**

The agar‐based culture of *Arabidopsis* seedlings is widely used for quantifying root traits. Shoot traits are generally overlooked in these studies, probably because the rosettes are often askew. A technique to assess the shoot surface area of seedlings grown inside agar culture dishes would facilitate simultaneous root and shoot phenotyping.

**Methods:**

We developed an image processing workflow in Python that estimates rosette area of *Arabidopsis* seedlings on agar culture dishes. We validated this method by comparing its output with other metrics of seedling growth. As part of a larger study on genetic variation in plant responses to nitrogen form and concentration, we measured the rosette areas from more than 2000 plate images.

**Results:**

The rosette area measured from plate images was strongly correlated with the rosette area measured from directly overhead and moderately correlated with seedling mass. Rosette area in the large image set was significantly influenced by genotype and nitrogen treatment. The broad‐sense heritability of leaf area measured using this method was 0.28.

**Discussion:**

These results indicated that this approach for estimating rosette area produces accurate shoot phenotype data. It can be used with image sets for which other methods of leaf area quantification prove unsuitable.

Automated image analysis techniques enable the non‐destructive phenotyping of large plant diversity panels. The 1001 Genomes Project is one example of such a panel; it comprises 1135 sequenced natural accessions of *Arabidopsis thaliana* (L.) Heynh. sampled from a wide range of environments (1001 Genomes Consortium, 2016). Combining these high‐quality genetic resources with high‐throughput phenotyping methods enables powerful genome‐wide association studies.

One technique for evaluating the developmental traits of such large diversity panels is growing the accessions in agar‐filled culture dishes. This allows root traits to be quantified quickly using high‐throughput image analysis methods. The plants are not destroyed or contaminated in the process and can therefore be photographed at different stages of growth. One disadvantage of this approach is that the rosettes are askew, so rosette area is usually not assessed even when the leaves are visible in the photographs. Quantifying both root and shoot characteristics is usually preferable because many plant processes involve both organs; for example, nitrogen acquisition and allocation involves root uptake from the rhizosphere, assimilation into organic forms in both the roots and shoots, and translocation throughout the plant (Bloom, 2015). Studying this process requires precise measurements of both the roots and shoots, which has previously been technically difficult.

Here, we show that leaf area measured from plate images is accurate even when the rosettes are somewhat askew and can therefore be used for rapidly phenotyping large image sets of *Arabidopsis* seedlings. As part of a larger study to examine the genetic basis of plant adaptation to different nitrogen forms and concentrations in the rhizosphere (Katz et al., 2022), we measured leaf area from more than 2000 images of *Arabidopsis* seedlings on agar plates. Our results demonstrate that this measurement is reproducible and provides a useful metric of shoot growth.

## METHODS

### Image sets for validating the accuracy of rosette area measurements from agar plate images

To determine whether rosette area measurements taken from plate images are sufficient for shoot phenotyping, we compared them to both measurements from images of the rosettes photographed from directly overhead and seedling mass.

To compare the overhead and plate image rosette area measurements, six different natural *Arabidopsis* accessions were planted on agar plates containing a base nutrient solution consisting of 2 mM CaCl_2_, 2 mM KH_2_PO_4_, 2 mM MgSO_4_, 1 mM KCl, 0.75 mM MES, 0.5 µM CuSO_4_, 2 µM MnSO_4_, 25 µM H_3_BO_3_, 42 µM FeNaDTPA, 2 µM ZnSO_4_, 0.5 µM H_2_MoO_4_, and 0.8% (w/v) agar. Different concentrations of sucrose (ranging from 0% to 2%) were added to the base media to ensure that there would be a variety of different‐sized seedlings. After planting, the plates were kept at 4°C for four days and then placed into a growth chamber with a 14‐h day/10‐h night cycle. After 12 days of growth, rosette area of the plants was measured in two ways, first from photographs of the seedlings in the plates and second from a photograph of the rosettes placed upright on paper. All photographs from this image set were taken with a Pixel 3A cellphone camera (Google, Mountain View, California, USA). A total of 58 seedlings were grown and measured this way.

As part of a larger study investigating plant responses to different nitrogen forms and concentrations in the rhizosphere, we quantified the rosette area from plate images and compared it with seedling mass. A total of 148 Col‐0 seedlings were grown under 10 different nitrogen conditions with either nitrate or ammonium as the sole nitrogen source at concentrations ranging from 0.05 mM to 5 mM. After 12 days of growth, the plates were photographed and the seedlings, including both roots and shoots, were excised and weighed.

### Image set from the *Arabidopsis* diversity panel study

As another part of the aforementioned study, more than 2000 images of *Arabidopsis* seedlings on agar plates were collected. This image set was generated from an experiment in which the 1135 natural accessions of the 1001 Genomes Project (1001 Genomes Consortium, 2016) were grown under four different nitrogen conditions: 0.1 mM and 1 mM nitrate using KNO_3_ as the sole nitrogen source and 0.1 mM and 1 mM ammonium using NH_4_HCO_3_ as the sole nitrogen source. The seedlings were grown under long‐day conditions (16 h light, 8 h dark). The closed plates were photographed 12 days after planting using an EOS Rebel digital camera fitted with an 18–55 mm EF‐S lens (Canon, Tokyo, Japan). The root traits, including primary root length and number of lateral roots, were estimated from the images using RootNav, image analysis software that allows the semiautomated quantification of complex root system architectures (Pound et al., 2013).

### Image analysis with Python

Some of the image sets did not have a red two‐dimensional scale present, making them unsuitable for rosette area measurement using existing methods such as Easy Leaf Area (Easlon and Bloom, 2014). We developed our own image processing workflows in Python, which were able to use a scale if it was present or, alternatively, to detect the area of the agar plate to serve as a scale. These workflows use the PlantCV package (Gehan et al., 2017) for most of the image‐processing functions.

**Figure 1:**
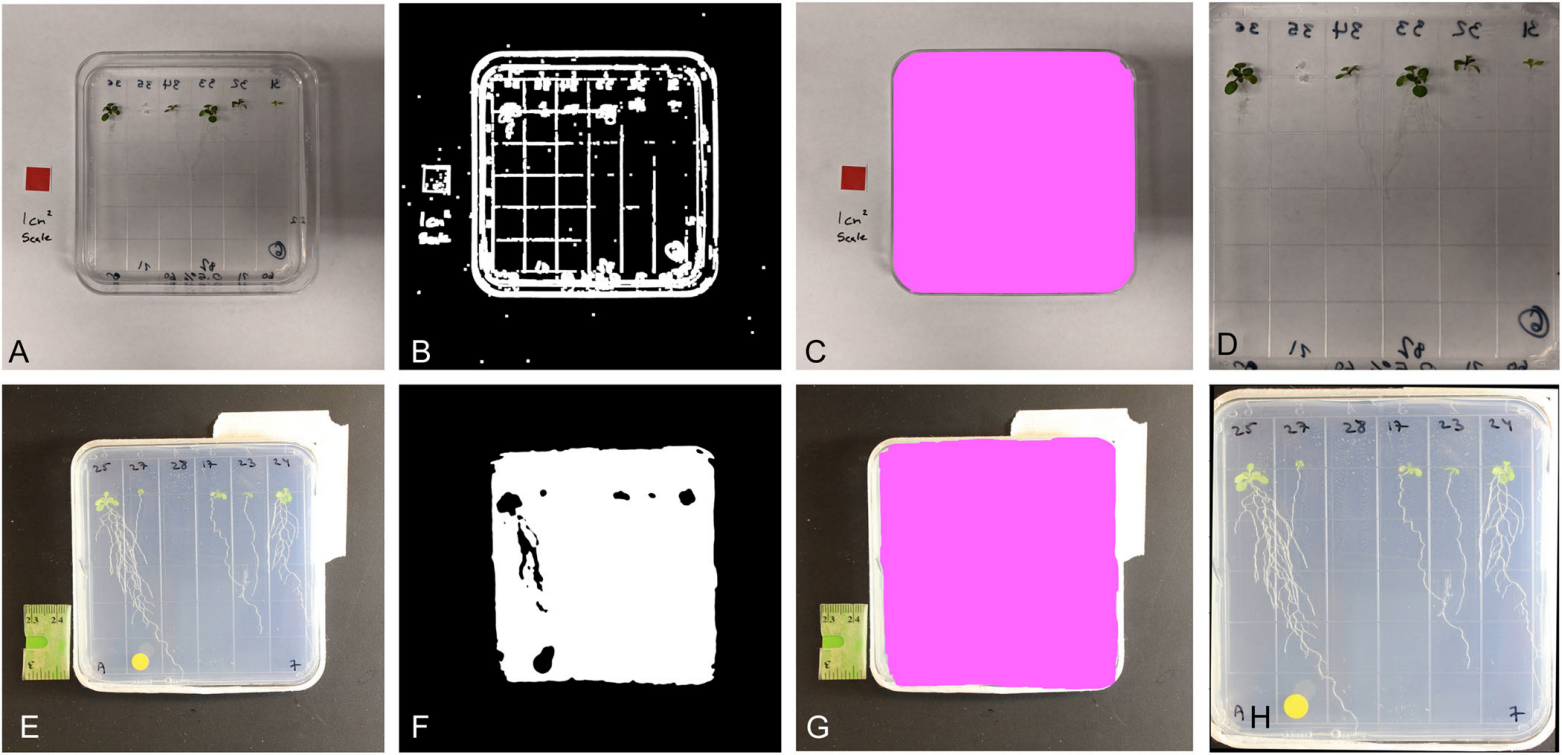
Two different image‐processing strategies used to identify the agar plate and automatically crop the image to the plate region. (A–D) Edge detection method. (A) Original image. (B) Binary image generated using canny edge detection. (C) Detection of plate area after the filtering steps. (D) The image after cropping to the plate region. (E–H) Thresholding detection method. (E) Original image. (F) Binary image generated using thresholding. (G) Detection of plate area after the filtering steps. (H) The image after cropping to the plate region and rotating.

The general steps in the workflow are (1) cropping the image to the plate region, (2) leaf identification and pixel counting, and (3) scale identification. Cropping the image to the region of interest (ROI) was done to save processing time and eliminate background features that could be mistaken for objects of interest. This was done using binary thresholding or edge detection to separate the agar‐filled culture dish from the background (Figure 1). The choice to use edge detection to identify the plate versus binary thresholding was dependent on the image set used. The detection of the agar plate also allows for the rotation of the image if the plate is not correctly aligned within the image.

**Figure 2:**
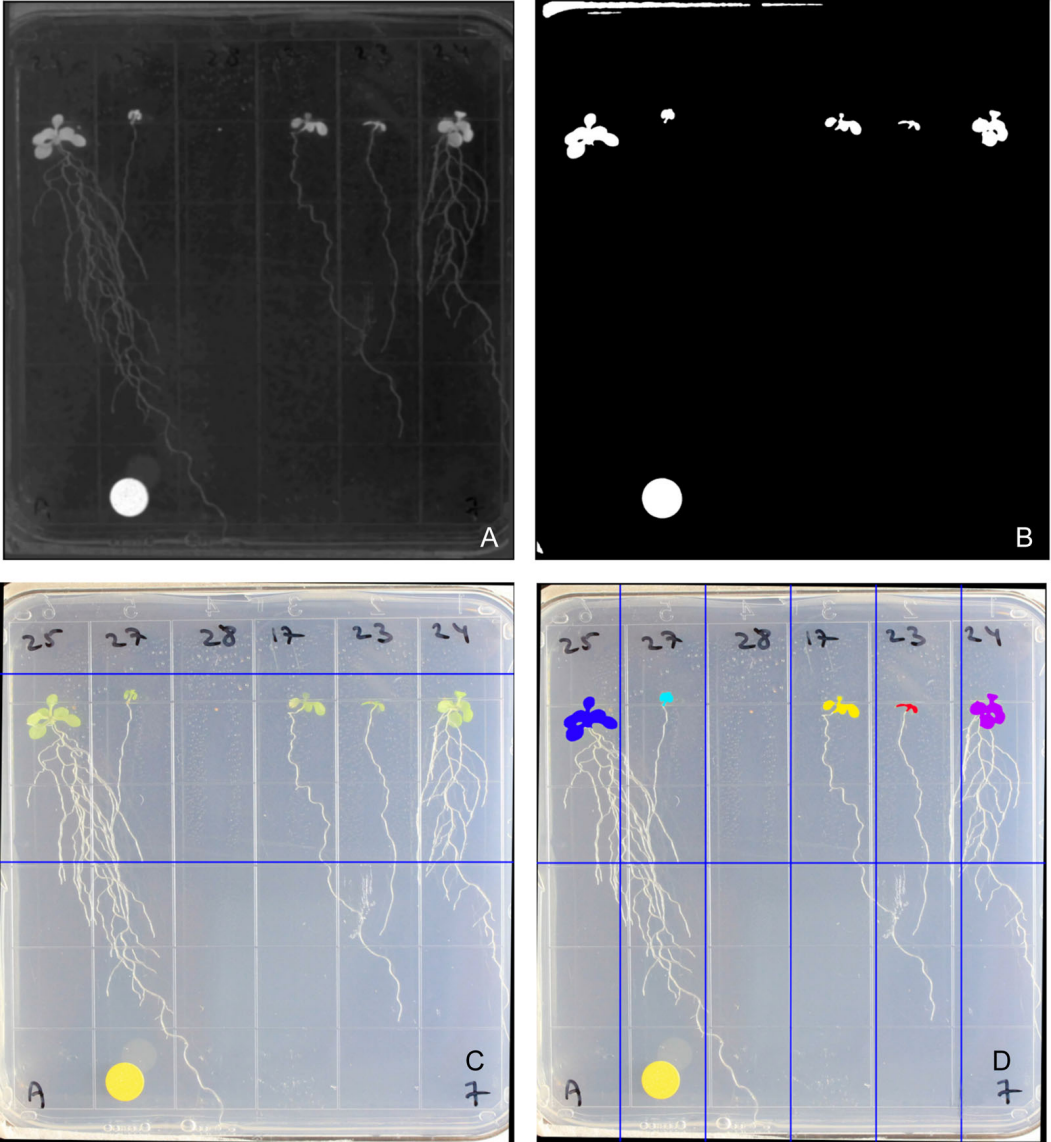
The image‐processing steps used for leaf identification. (A) Image converted to black and white using the B channel of the L*a*b* color space. (B) Binary threshold. (C) Region of interest indicated by the blue lines. (D) Identification of individual shoots using clustering and splitting the image into six sections. Note that one seed failed to germinate so no leaf area was measured.

Leaf identification was performed using binary thresholding and object detection (Figure 2). The specific color channel and threshold value used to identify the leaves varied between the different image sets due to different background and lighting conditions, but as long as the images within a set are taken against the same background and with the same lighting conditions then these values should remain consistent for processing the entire set. For the validation images, the “C” channel of CMYK color space was used to identify the leaves, whereas in the diversity panel image set the “B” channel of L*a*b* color space was used. To determine the appropriate threshold values for an image set, we used the plot histogram function in PlantCV. This function is used to visualize the range of pixel intensity in the color channel of interest. For image sets with lower contrast, the histogram equalization function was used to make thresholding easier. To simplify leaf identification, an ROI was defined for the top section of the cropped image where the leaves are found. Objects detected within the ROI were grouped into six shoots using clustering. The image moment of each shoot in the binary image was used to calculate the number of pixels that made up the leaves in each seedling.

Scale identification was performed either by using a reference scale that was placed within the image or using the plate itself as a scale. For dedicated reference scales, the same general process that was used to identify and count pixels of the leaves was used for the scale. Once the number of pixels in the scale or the number of pixels making up the plate were measured, the rosette area could be calculated.

For the large image set of the diversity panel, we automated the workflow in a Python script, which took approximately four hours to process all 2000 images. We were able to estimate rosette area for over 90% of the seedlings that successfully germinated, resulting in 8964 individual measurements. Many of the seedlings that were not measurable had fallen below the middle of the plate and were not within the defined ROI.

The workflows for each image set can be found at https://github.com/massivejords/Agar-plate-leaf-area as Jupyter Notebooks (Kluyver et al., 2016), along with the batch analysis Python script used to process the large image set. It is important to note that the parameters used for the various transformations, such as thresholding, grayscale conversion, and scale calculation, are specific to the image set. These parameters would need to be modified when using a different image set, but the general steps would still apply.

### Statistics and data visualization

Linear least‐squares regressions were calculated for the validation data sets using Microsoft Excel (Microsoft, Redmond, Washington, USA). R software (R Core Team, 2022) with the RStudio interface (http://www.rstudio.com/) and the tidyverse (Wickham et al., 2019) and ggplot2 (Wickham, 2016) packages was used to analyze and visualize the data from the diversity panel study. We performed an ANOVA to test the effect of genotype, nitrogen source, and nitrogen treatment (nitrogen source and concentration) on the seedling rosette area and to determine the broad sense heritability of the trait.

## RESULTS

### Validation

**Figure 3:**
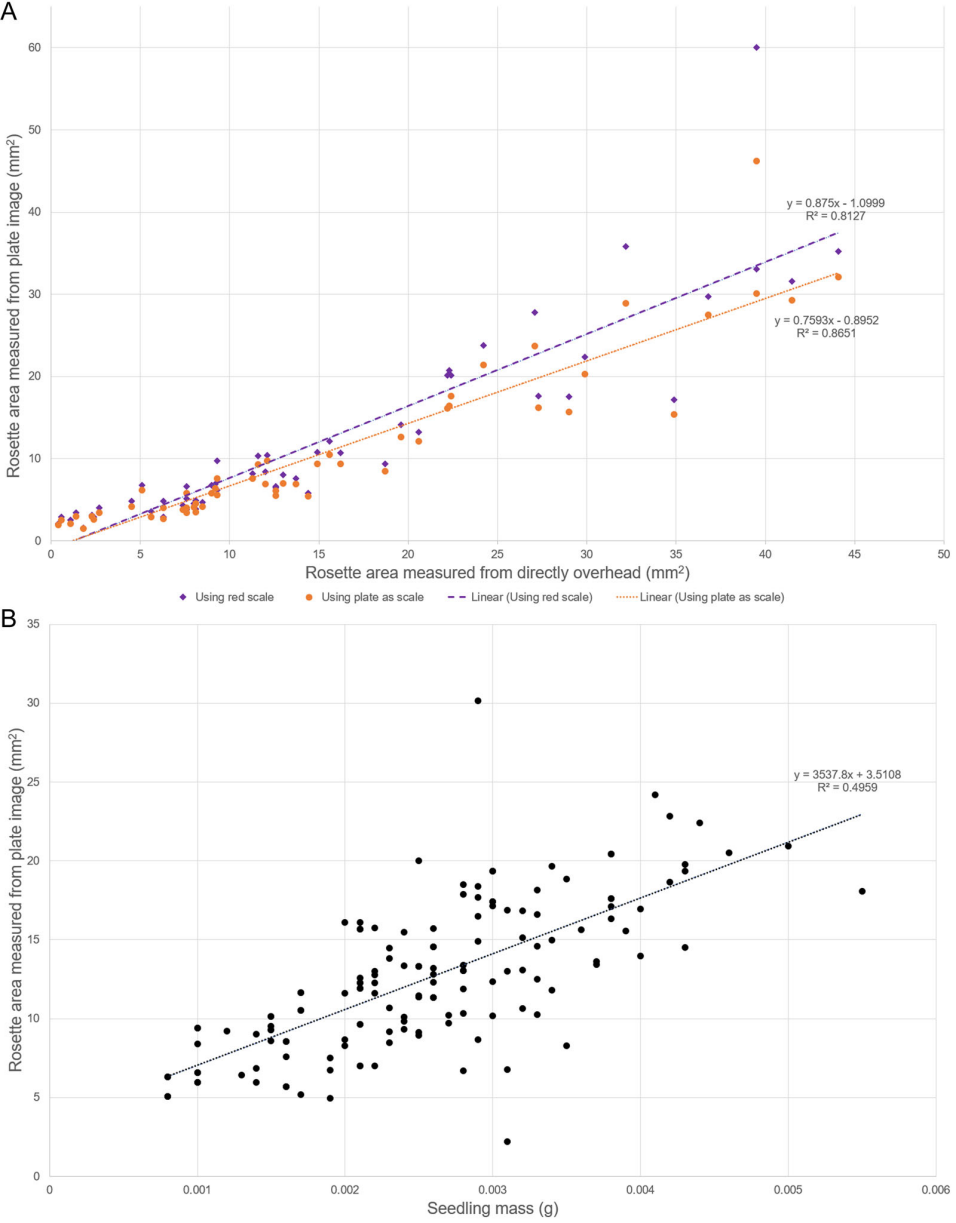
Scatterplots of rosette area measured from agar plate images compared with other metrics of growth. Dashed lines indicate linear least‐squares regression fits to the data points. (A) Scatterplot comparing *Arabidopsis* seedling rosette areas measured from agar plates or directly overhead. (B) *Arabidopsis* seedling rosette area measured from agar plates compared with seedling mass.

Rosette area measurements taken from plate images and from directly overhead were found to have a strong linear relationship (Figure 3A). Rosette areas from this image set were quantified twice, first using the red square as a scale and then using the plate area as a scale. For each set of measurements, a linear least‐squares regression was calculated. The coefficient of determination (*R* ^2^) for measurements that relied on the red scale was 0.8127, and the slope and *y* intercept were 0.875 and −1.100, respectively. For plate image measurements that used the plate itself as a scale, *R* ^2^ was 0.8651, the slope was 0.759, and the *y* intercept was −0.895. Seedling mass and rosette area were also found to be positively correlated (Figure 3B), although this correlation was not as strong (*R* ^2^ = 0.4959, slope = 3537.8, *y* intercept = 3.5).

### Rosette area response to nitrogen nutrition in an *Arabidopsis* diversity panel

**Figure 4:**
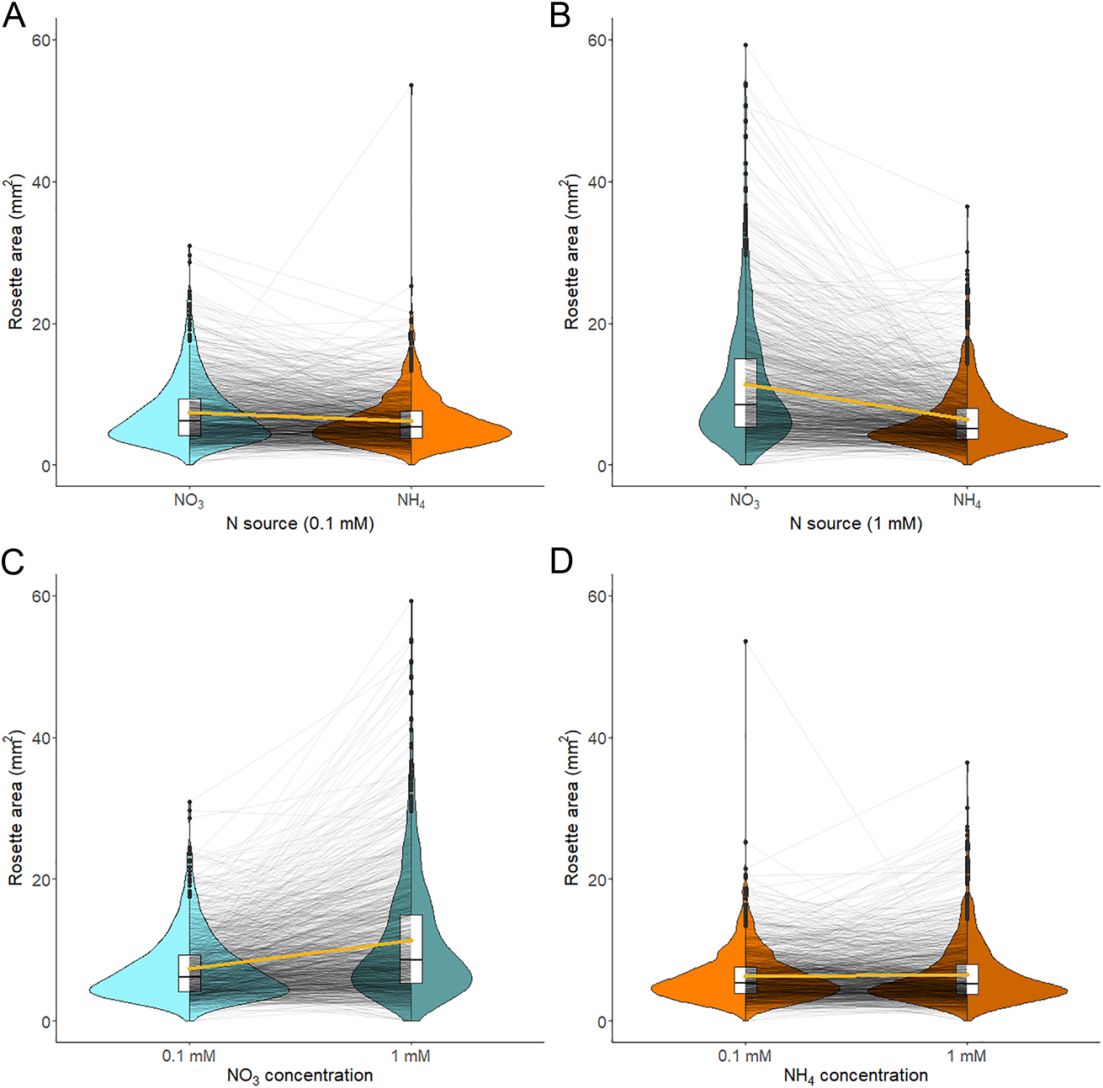
Leaf area of *Arabidopsis* seedlings under different nitrogen nutrition treatments. Black lines connect leaf area of the same accession under different treatments and the gold lines connect the mean for each treatment. Ammonium treatments are indicated in orange and nitrate treatments in blue; lighter shades indicate a concentration of 0.1 mM and darker shades indicate 1 mM. (A) Response to the nitrogen forms at a concentration of 0.1 mM. (B) Response to the nitrogen forms at a concentration of 1 mM. (C) Response to nitrate concentration. (D) Response to ammonium concentration.

The response of rosette area to the four nitrogen nutrient conditions across the 1135 accessions is shown in Figure 4. Overall, the seedlings grown in 1 mM nitrate had the greatest mean rosette area. Some accessions showed extreme differences in their responses to the nitrogen conditions. Nitrogen source and treatment both had significant effects on rosette area (*F*_1,5360_ = 547.62, *P* < 0.0001 and *F*_2,5360_ = 238.94, *P* < 0.0001, respectively). Genotype also had a significant effect (*F*_1020,5360_ = 3.70, *P* < 0.0001), and the percentage of the total sum of squares attributed to this factor was used to calculate an *H* ^2^ of 0.28. The heritability of primary root length was 0.41.

## DISCUSSION

The strong positive linear relationship between the rosette area measurements taken from the plate images and those taken from photographs of excised rosettes demonstrates that using plate images for shoot trait analyses can yield meaningful phenotype data with minimal effort. While the correlation between rosette area measured from plate images and seedling mass was not as strong, it was still sufficient to indicate that this is a viable method for estimating plant growth. A lower correlation between these measurements is also to be expected because the seedling mass includes both shoot and root mass and is therefore not as specific to shoot growth as is the rosette area.

We were also able to apply this analysis to an image set generated for the purpose of root phenotyping, allowing us to obtain additional valuable phenotypic information. The rosette area measured using this technique across a large *Arabidopsis* diversity panel was found to be heritable and showed a significant response to rhizosphere nitrogen form and concentration. These results were in line with other developmental traits measured using established techniques, such as primary root length measured using RootNav (Pound et al., 2013).

Agar plate images are widely used for the non‐destructive measurement of *Arabidopsis* root traits. Here, we showed that useful shoot trait information can also be collected from these same images, enabling simultaneous root and shoot phenotyping. This can be done quickly and is easily automated, making it suitable for large image sets. The images can be captured and analyzed without the need for specialized imaging equipment or dedicated phenotyping facilities. The agar plate itself can be used as a scale, enabling the analysis of image sets without dedicated two‐dimensional scales. With the procedures described here, image sets generated for root phenotyping in other studies might also provide data about shoot phenotypes without much additional effort.

## AUTHOR CONTRIBUTIONS

J.S. and A.K. wrote the software and performed the validation under supervision from E.K. and A.J.B. E.K. provided resources required to validate this method. A.K. prepared the original draft, and A.J.B and E.K. edited and revised it. All authors approved the final version of the manuscript.

## Data Availability

All of the software described in this paper is available on GitHub at https://github.com/massivejords/Agar-plate-leaf-area.
